# Supporting postgraduate exam preparation with large language models: implications for traditional Chinese medicine education

**DOI:** 10.3389/fmed.2025.1667104

**Published:** 2026-01-09

**Authors:** Baifeng Wang, Meiwei Zhang, Zhe Wang, Keyu Yao, Meng Hao, Junhui Wang, Suyuan Peng, Yan Zhu

**Affiliations:** 1 Dongzhimen Hospital, Beijing University of Chinese Medicine, Beijing, China; 2 School of Medical Informatics, Changchun University of Traditional Chinese Medicine, Changchun, China; 3 Institute of Medical Informatics, Statistics,and Epidemiology,Leipzig University, Leipzig, Germany; 4 Institute of Information on Traditional Chinese Medicine, China Academy of Chinese Medical Sciences, Beijing, China; 5 Guang'anmen Hospital,China Academy of Chinese Medical Sciences, Beijing, China

**Keywords:** large language models (LLMs), traditional Chinese medicine, medical education, Ernie Bot, ChatGLM, SparkDesk, GPT-4

## Abstract

**Introduction:**

In China, the medical education system features multiple co-existing levels, with higher education often leading to better job prospects. In career advancement—especially for entry into competitive urban hospitals—the postgraduate examination often plays a more decisive role than the licensing examination. The application of Large Language Models (LLMs) in Traditional Chinese Medicine (TCM) has rapidly expanded. TCM theories possess distinct scientific features, requiring LLMs to demonstrate advanced information processing and comprehension abilities in a Chinese context. While LLMs have shown strong performance in many countries' licensing examinations, their performance in selective TCM examinations remains underexplored. This study aimed to evaluate and compare the performance of Ernie Bot, ChatGLM, SparkDesk, and GPT-4 on the 2023 Chinese Postgraduate Examination for TCM (CPE-TCM), and explore their potential in supporting TCM education and academic development.

**Methods:**

We assessed the performance of four LLMs using the 2023 CPE-TCM as a test set. Exam scores were calculated to evaluate subject-specific performance. Additionally, responses were qualitatively analyzed based on logical reasoning and the use of internal and external information.

**Results:**

Ernie Bot and ChatGLM achieved accuracy rates of 50.30 and 46.67%, respectively, both above the passing score. Statistically significant differences in subject-specific performance were observed, with the highest scores in the medical humanistic spirit module. ChatGLM and GPT-4 provided logical explanations for all responses, while Ernie Bot and SparkDesk showed logical reasoning in 98.2 and 43.6% of responses, respectively. ChatGLM and GPT-4 incorporated internal information in all explanations, whereas SparkDesk rarely did. Over 60% of responses from Ernie Bot, ChatGLM, and GPT-4 included external information, which did not significantly differ between correct and incorrect answers. In SparkDesk, the presence of internal or external information was significantly associated with answer correctness (*P* < 0.001).

**Discussion:**

Ernie Bot and ChatGLM surpassed the passing threshold for postgraduate selection, reflecting solid TCM expertise. LLMs demonstrated strong capabilities in logical reasoning and integration of background knowledge, highlighting their promising role in enhancing TCM education.

## 1 Introduction

Physicians in most high-income countries undergo similar levels of educational training and share a homogeneous academic background (1). In response to the demand for healthcare, China has developed a complex medical education system aimed at increasing the number of physicians. Furthermore, the criteria for graduation and employment of medical doctors vary. Doctors with lower levels of education mainly practice in townships and rural areas in China, while those with higher levels of education are more likely to work in urban areas (1). Within the current system, there is a 3-year junior college medical program, a 5-year Bachelor of Medicine degree program, a “5+3” Master of Medicine degree program, and an 8-year Doctor of Medicine program (2). The medical profession has established degree levels to meet the demand for professional healthcare and ensure the provision of basic and primary medical services to the public (3). Despite undergoing multiple reforms and advancements, there remains a significant gap between the medical education system and elite medical education.

A growing number of undergraduate students in China are now pursuing advanced degrees like master's or doctoral programs (2). In the past 3 years, over four million candidates have taken Chinese Postgraduate Examination (CPE) annually. Recent data on postgraduate enrollment showed medicine is among the top five disciplines in terms of enrollment size, and it had the highest growth rate in 2021 (4). As of the end of 2020, 59.5% of the physicians in the workforce had a bachelor's degree or higher.

Within China's multi-tiered medical education and practitioner system, the National Medical Licensing Examination (NMLE) serves as the mandatory gateway for clinical practice. However, for career advancement and entry into competitive urban hospitals, CPE is often the more critical and decisive step (5). Existing studies have primarily focused on medical licensing examinations, indicating that large language models (LLMs) may play a supportive role in medical education. However, it remains unclear whether LLMs can assist medical students in succeeding in competitive graduate entrance examinations and advancing their academic qualifications.

Significant advancements have been observed in Artificial Intelligence (AI) modeling in recent years, leading to the rapid development of LLM technology. Particularly noteworthy is the emergence of products like ChatGPT (OpenAI, 2022), signaling a new era for the deployment of general-domain AI (6). Currently, LLMs have demonstrated promising applications in various areas of medical research, including diagnostics (7), medical image analysis (8), medical writing (9), and personalized medicine (10).

The medical licensing examination is commonly used to assess the performance of LLMs in the medical field due to its high standardization, regulation, and comprehensive coverage of various subjects. Multiple studies have systematically evaluated ChatGPT's performance on standardized tests across various languages. Notably, it has demonstrated excellent performance on assessments such as the United States Medical Licensing Examination (USMLE) (11–13), the Japanese Medical Licensing Examination (JMLE) (14), the Saudi Medical Licensing Examination (SMLE) (15), the Polish medical specialization licensing exam (PES) (16) and Taiwan's medical licensing exams (17). However, over the past 5 years of the Chinese NMLE, ChatGPT scores have consistently fallen below the passing threshold. The main reason is that ChatGPT's training data is mainly in English, with only a small amount in Chinese. Additionally, ChatGPT may face challenges in accurately comprehending healthcare policies within non-English speaking nations (18, 19). Cai et al. introduced a comprehensive benchmark for the Chinese medical domain and evaluated both general-domain and medical LLMs in Chinese. The findings suggest that general-domain LLMs possess substantial medical knowledge and may perform better than medical LLMs (20).

However, most existing evaluations remain focused on modern biomedicine. Research on LLM performance in traditional medical systems—such as Traditional Chinese Medicine (TCM) or Traditional Korean Medicine—is still scarce, despite their unique theoretical and linguistic characteristics.

The application of LLMs in the education and assessment of East Asian traditional medicine has been gradually expanding. Previous studies have evaluated the performance of GPT-4 on Korea's national licensing examination for Traditional Korean Medicine (21) and examined ChatGPT-4 on Taiwan's TCM physician licensing examination (22). Both studies showed that LLMs could handle standardized questions to some extent but still lacked explanatory depth and higher-order reasoning. These studies, however, focused on licensure-oriented exams that emphasize minimum clinical competency, regulatory compliance, and knowledge breadth.

In contrast, the present study adopts the Chinese Postgraduate Examination for TCM (CPE-TCM), which is an academically selective examination. Its evaluation focus shifts from assessing “minimum competency” to higher-level cognitive abilities. The design of the test places greater emphasis on knowledge depth, integration, and differentiation, aiming to comprehensively assess candidates' understanding of foundational TCM theories, clinical reasoning, and the ability to transfer knowledge across contexts. It represents a cognitively challenging task with a high degree of discrimination. Notably, in addition to standard single-choice questions (A-type and B-type), the exam also includes highly discriminative multiple-choice questions (X-type). These require examinees to accurately grasp and horizontally integrate multiple knowledge points, imposing significantly greater cognitive demands than those of typical licensing examinations. The exam content also focuses more specifically on core disciplines of TCM, excluding non-essential topics such as basic Western medicine and medical laws and regulations, which are commonly included in licensure exams. Therefore, CPE-TCM provides a more sensitive evaluation scenario for assessing the depth of understanding and the ability of LLMs to integrate domain-specific knowledge. In addition to these distinctions, data accessibility also influenced our choice of examination. Although the NMLE is equally authoritative, its complete and verified question sets are not publicly available, and online items lack authenticity. By contrast, CPE-TCM offers a legally published, standardized, and fully accessible corpus, ensuring reliable and reproducible evaluation.

Compared with previous studies that used physician or pharmacist licensing examinations, this study offers a new academic perspective by systematically evaluating the comprehension and reasoning abilities of LLMs in the context of advanced TCM education. This design not only expands the scope of education-oriented assessment tasks but also lays a theoretical foundation for future applications of LLMs in postgraduate education, instructional support, and personalized learning in TCM.

TCM is a comprehensive medical system rooted in centuries of clinical experience and theoretical development in China. It plays an important role in disease prevention and treatment, and its global impact is growing, with acupuncture formally recognized in over 100 WHO member states. Given its distinctive theoretical framework and contextual reasoning features, systematic evaluation of LLMs in answering TCM-related questions is necessary to ensure their reliability and applicability in this specialized domain.

Therefore, this study aims to systematically evaluate the performance of mainstream LLMs on CPE-TCM under simulated test conditions and to explore their potential in supporting postgraduate TCM education. This benchmark offers several advantages: it (i) comprehensively covers core TCM subjects; (ii) is authored by domain experts, ensuring content validity; (iii) provides highly standardized items with verifiable answers; and (iv) supplies official scoring anchors (i.e., passing thresholds).

## 2 Methodology

### 2.1 Models

In addition to the globally recognized GPT-4 (6), we selected three representative Chinese LLMs—Ernie Bot, ChatGLM, and SparkDesk—to enhance language adaptation and methodological coverage. Ernie Bot is built upon the ERNIE architecture, which adopts a knowledge-enhanced pre-training paradigm by integrating structured knowledge with large-scale corpora (23). It has demonstrated strong performance across a range of Chinese NLP tasks and is particularly suitable for addressing structured, knowledge-dependent professional questions as featured in this study. ChatGLM, derived from the GLM framework, employs an autoregressive blank infilling objective to support a unified pre-training architecture (24). It emphasizes architectural generalization and task compatibility, providing a solid foundation for logical reasoning and option-level analysis. SparkDesk prioritizes education-oriented deployment. It has been applied across multiple educational scenarios—including personalized learning, lesson planning, and classroom-integrated devices—demonstrating a high degree of engineering maturity and practical usability (25).

Collectively, these models represent three major technical pathways in Chinese LLM development—knowledge enhancement, unified architecture, and application-driven integration. Their inclusion enables a multi-faceted evaluation of LLM performance in the context of this study and provides a balanced basis for comparison with GPT-4.

All models were accessed via official web interfaces with browsing/plugins disabled and default inference settings (no manual parameter adjustments).

### 2.2 Medical examination datasets

CPE-TCM is a nationally administered selective examination organized and developed by the National Education Examinations Authority under the Ministry of Education of China. It is designed for all universities nationwide that enroll master's students in TCM programs, featuring a high degree of standardization and authority. Therefore, the examination questions reflect the core national requirements for the knowledge structure and competency level expected of candidates entering TCM postgraduate programs. The test content focuses closely on classical TCM theories and core clinical disciplines, and is widely regarded within the TCM education system as an important indicator of undergraduate learning outcomes and postgraduate training potential.

We collected questions from the CPE-TCM in 2023, for a total of 165 questions. The examination comprised single-choice and multiple-choice questions spanning seven distinct disciplines: Basic Theory of TCM, Diagnostics of TCM, Pharmacology of TCM, Formulas of TCM, Internal Medicine of TCM, Acupuncture and Moxibustion, and Medical Humanistic Spirit (Table 1).

**Table 1:** Subjects examined in the test questions.

**Subjects**	**Number of problems**	**Examples**
Basic theory of TCM	23	According to mutual generation and control among the five elements, the principle of restraining the strong and supporting the weak is demonstrated by () A. Warming the kidney and tonifying the spleen B. Tonifying the kidney and enriching the liver C. Purging the heart and clearing the liver D. Soothing the liver and tonifying the spleen
Diagnostics of TCM	23	What is not a common indication of bluish and black facial complexion? A. Blood stasis pattern, pain B. Blood stasis pattern, infantile convulsions C. Cold pattern, pain D. Cold pattern, blood stasis pattern
Pharmacology of TCM	23	According to the theory of medicinal properties, most of the drugs used to treat edema and inhibited urination have the flavor of () A. Astringent flavor B. Bland flavor C. Salty flavor D. Bitter flavor
Formulas of TCM	23	The drug found in both Qianghuo Shengshi Decoction and Daqinjiao Decoction is (). A. Atractylodis rhizoma and Angelicae pubescentis radix B. Chuanxiong rhizoma and Saposhnikoviae radix C. Ligustici rhizoma et radix and Angelicae dahuricae radix D. Asari radix et rhizoma and Glycyrrhizae radix et rhizoma
Internal medicine of TCM	43	A 34-year-old woman with a 3-month history of coughing showed increased texture in the bilateral lower lungs on a chest X-ray. Coughing, distension in the sub-costal region, dry throat and a bitter taste, scanty and sticky phlegm, red tongue with thin, yellow coating, and wiry pulse, belong to () A. Wind dryness damaging the lung pattern B. Lung yin deficiency pattern C. Liver fire affecting the lung pattern D. Lung heat and yin deficiency pattern
Acupuncture and moxibustion	22	Located seven inches below the cubital crease is () A. LU6 B. LI6 C. PC4 D. SJ6
Medical humanistic spirit	8	The medical profession is the community that is accountable for the commitments expected by the public, and the following narrative is incorrect () A. The medical profession does not require systematic training. B. Professional community self-regulation is essential for the medical profession. C. The medical profession earns community trust by enforcing industry standards. D. Professional integrity within the medical profession is upheld through a rigorous admission system.

### 2.3 Prompt engineering

The prompts have a significant impact on the output of LLMs (26). To obtain the best form and content of answers, we supplied prompts and instructed LLMs to furnish explanations and justifications for their selected answers.

Each question was input in the specified format: “The following is a single-choice question from the Chinese Comprehensive Ability of Clinical Medicine (TCM) Examination. Please choose the correct answer and provide a correct and reasonable answer analysis” (single-choice question) or “The following is a multiple-choice question from the Chinese Comprehensive Ability of Clinical Medicine (TCM) Examination. For each sub-question, there are four options labeled A, B, C, and D, among which at least two meet the requirements of the question. Please choose the correct answers and generate a correct and reasonable answer analysis” (multiple-choice question). Next, input the question and its corresponding options. Table 2 displays an example question along with the answers provided by the LLMs.

**Table 2:** Response scenarios for the identical question from Ernie Bot and GPT-4.

**Input problem**	**Ernie Bot**	**GPT-4**
The following is a single-choice question from the Chinese Comprehensive Ability of Clinical Medicine (TCM) Examination. Please choose the correct answer and provide a correct and reasonable answer analysis. A 49-year-old female presenting with insomnia persisting for 4 months, compounded by vexation with insomnia, difficulty in falling asleep, palpitation and dreaming with dizziness and tinnitus, along with low back soreness and knee weakness, tidal fever, night sweats, feverish sensations in palms, soles and chest, dry throat with little fluids, accompanied by a red tongue displaying scant coating and thready, rapid pulse, is suitable for (). A. Zuojin Wan and Suanzaoren Decoction B. Zhibai Dihuang Wan C. Liuwei Dihuang Wan and Jiaotai Wan D. Tianma Gouteng Decoction	The correct answer is B. Zhibai Dihuang Wan The patient, a middle-aged woman, is experiencing insomnia, compounded by vexation with insomnia, difficulty in falling asleep, palpitations, dreaminess, dizziness, and tinnitus. Additionally, she is struggling with low back soreness and knee weakness, tidal fever and night sweats, feverish sensations in palms, soles and chest, as well as dry throat with little fluids, a red tongue with scant coating and a thready, rapid pulse. After assessing her symptoms, tongue manifestations, and pulse conditions, she was diagnosed with insomnia caused by yin deficiency leading to fire hyperactivity. The treatment aims to nourish and tonify the blood and tranquilize the spirit. The formula is a modified version of Suanzaoren Decoction (A is incorrect). This question also includes symptoms of a dry throat and little fluids. The Suanzaoren Decoction does not include the treatment of nourishing yin and clearing the lungs to benefit the throat, so it is excluded. The patient's insomnia, attributed to yin deficiency leading to fire hyperactivity, warrants treatment focusing on heart and kidney perspectives. Coptidis Rhizoma in Zuojin Wan and Suanzaoren Decoction is specially formulated to clear the heart and tranquilize the spirit, effectively treating insomnia. However, in this case, kidney yin deficiency is predominant and treatment should be based on nourishing and supplementing kidney yin. So Zuojin Wan is not appropriate (A is incorrect). The patient's insomnia is caused by yin deficiency leading to fire hyperactivity. Treatment should focus on nourishing and tonifying the blood to tranquilize the spirit. The patient's symptoms are mainly low back soreness and knee weakness, dizziness and tinnitus, tidal fever and night sweats, feverish sensations in palms, soles and chest, dry throat and little fluids, red tongue with little coating, and a thready and rapid pulse. These indicate insomnia resulting from yin deficiency leading to fire hyperactivity. Liuwei Dihuang Wan is formulated to tonify the kidney. However, since kidney-yin deficiency predominates in this case, treatment should prioritize nourishing and supplementing kidney-yin. Therefore, Liuwei Dihuang Wan is not suitable (C is incorrect). Tianma Gouteng Decoction is weak in smoothing the liver, submerging yang, nourishing blood, and calming the mind. It is not effective in treating insomnia caused by yin deficiency leading to fire hyperactivity (D is incorrect). Zhibai Dihuang Wan is effective in enriching yin and reducing fire, making it suitable for treating insomnia caused by yin deficiency leading to fire hyperactivity (B is correct).	The correct answer is: B. Zhibai Dihuang Wan Answer analysis: Based on the patient's symptoms including insomnia, palpitations, dreaminess, dizziness, tinnitus, low back soreness and knee weakness, tidal fever, night sweats, feverish sensations in palms, soles, and chest, dry throat with little fluids, as well as tongue and pulse observations (red tongue with scanty coating and thready, rapid pulse), the pattern corresponds to insomnia resulting from yin deficiency leading to fire hyperactivity. Zhibai Dihuang Wan, a classic formula for nourishing yin and reducing fire, is primarily prescribed to alleviate symptoms like tidal fever, night sweats, feverish sensations in the palms, soles, and chest, and dry mouth resulting from kidney-yin deficiency. A. Zuojin Wan combined with Suanzaoren Decoction is primarily employed to address insomnia resulting from the transformation of qi stagnation into fire. C. Liuwei Dihuang Wan combined with Jiaotai Wan is primarily utilized to treat symptoms arising from kidney-yin deficiency and kidney-yang deficiency. However, since the main symptom of the patient is yin deficiency leading to fire hyperactivity, Jiaotai Wan, being warm, is not suitable for this pattern of fire hyperactivity. D. Tianma Gouteng Decoction is mainly used for the internal stirring of liver wind pattern. Therefore, considering the patient's symptoms, the most appropriate option is Zhibai Dihuang Wan. It effectively enriches yin and reduces fire, making it suitable for treating insomnia resulting from yin deficiency leading to fire hyperactivity.

Both the prompts and the test items were presented to all models in their original Chinese form, and all responses were generated entirely based on Chinese text. The English examples shown in the manuscript are human translations provided after the evaluation for illustrative purposes only; they were not used in model inference and had no influence on model performance or evaluation outcomes.

### 2.4 Data analysis

All model testing was conducted by inputting questions and obtaining answers provided by the LLMs. All responses were recorded in an electronic spreadsheet, and the chosen answers from each response were extracted and compared to the standard answers. Each question was considered “correct” only if the answer choices matched the standard answer, whereas incorrect choices, missing options, or ambiguous responses were labeled as “incorrect.” The accuracy of LLMs' responses was calculated after the process.

The scoring system for the question set was structured as follows (Table 3):

**Table 3:** Scoring structure.

**Question type**	**Examined domains**	**Question range**	**Points/question**	**Subtotal**
**Single-choice**				
Phase 1	Foundational knowledge^†^	1–36	1.5	54
Phase 2	Clinical & humanities^‡^	37–81	2.0	90
Phase 3	Integrated synthesis	82–105	1.5	36
**Multiple-choice**				
	Integrated synthesis	106–165	2.0	120
Total				300

^†^Foundational knowledge: basic theory, diagnostics, pharmacology, formulas.

^‡^Clinical & humanities: internal medicine, acupuncture & medical humanistic spirit.

Single-choice questions progress across three phases: foundational knowledge (Phase 1), clinical knowledge with humanities (Phase 2), and interdisciplinary integration (Phase 3), each calibrated with weighted points reflecting cognitive demands. Multiple-choice questions extend this framework to assess advanced problem-solving in complex scenarios. Differential scoring (1.5–2.0 points/question) emphasizes clinical reasoning and synthetic thinking, ensuring systematic evaluation of both specialized expertise and holistic TCM proficiency.

We calculated the accuracy rates of LLMs' responses across various subjects in the test questions and conducted statistical analysis on them. SPSS 27.0 software was used for data processing, and *Pearson's* χ^2^ test was used for comparison of rates, with *P* < 0.05 indicating a statistically significant difference. To compare within-model accuracy differences across disciplines (one omnibus χ^2^ test per model), we controlled the family-wise error rate (FWER) using the Bonferroni correction across the four primary tests (adjusted α = 0.05/4 = 0.0125). We report Bonferroni-adjusted *P* values. All tests were two-sided.

### 2.5 Qualitative analysis

Three binary variables were employed to assess the responsiveness of LLMs toward each question:

1. Logical reasoning: The response demonstrated the logical connection between the provided information and the answer choices.
2. Ability to use internal information: The response incorporated information relevant to the question, including analyses derived from case content or essential explanations focused on the question.
3. Ability to use external information: The response incorporated external information beyond the scope of the question, including but not limited to additional analyses and expansions of the topic, provided answers, and distractors.

Each incorrect answer was categorized as one of three types based on the reason for the mistake:

1. Logical error: The parsing contained relevant information but did not correctly convert it into answers. The options in the parsing differed from those in the output. The parsing contained contradictory statements.

Example Question: “What can cause low-grade fever?” Model output (GPT-4): “D. Qi stagnation: Qi stagnation is not directly related to low-grade fever, but prolonged Qi stagnation can transform into Fire, which can cause a low-grade fever.” The analysis showed that Qi stagnation could lead to a low-grade fever, but option D was not selected in the response. This is a logical error.

1. Information error: The extraction, interpretation, and application of internal information or the presentation of external information is incorrect.

Example Question: According to “Huang Di Nei Jing Su Wen,” what are the manifestations of men at “five times eight”? Model output (Ernie Bot): according to “Huang Di Nei Jing Su Wen,” men at “five times eight” exhibit strong tendons and bones (A is correct).” Due to this misinformation, the answer given is incorrect.

2. The presence of both types of errors concurrently.

This study involved three evaluators: two senior postgraduate students in TCM who successfully completed the CPE-TCM, and one senior clinical professional in TCM. To familiarize the evaluators with the scoring system, 30 questions from the 2022 CPE-TCM were used for training purposes. The process was independently assessed by the two masters based on the mentioned criteria and then cross-checked. In the event of disputes, another Chinese medicine professional would make the final judgment.

## 3 Results

### 3.1 Accuracy and scoring performance

The official passing threshold was 117 points. Ernie Bot, the highest achiever, attained a score of 146 by correctly answering 83 questions (50.30%). ChatGLM also successfully passed the exam with a score of 136.5, demonstrating an accuracy of 46.67%. However, SparkDesk and GPT-4 did not pass the examination, scoring 86 and 112 points, respectively.

### 3.2 Performance on different subjects

Table 4 summarizes the test results for each subject. After applying the Bonferroni correction across the four omnibus χ^2^ tests, only GPT-4 retained a statistically significant heterogeneity across disciplines (*P*_adj < 0.004). The heterogeneity for Ernie Bot (*P*_adj = 0.080), ChatGLM (*P*_adj = 0.104), and SparkDesk (*P*_adj = 1.000) was not statistically significant. All models involved in the test exhibited the highest accuracy rates in questions related to the medical humanistic spirit.

**Table 4:** The performance of LLMs on different subjects.

**Model**	**Overall *n* = 165, *n* (%)**	**Basic theory of TCM *n* = 23, *n* (%)**	**Diagnostics of TCM *n* = 23, *n* (%)**	**Pharmacology of TCM *n* = 23, *n* (%)**	**Formulas of TCM *n* = 23, *n* (%)**	**Internal medicine of TCM *n* = 43, *n* (%)**	**Acupuncture and moxibustion *n* = 22, *n* (%)**	**Medical humanistic spirit *n* = 8, *n* (%)**	***P* (Bonferroni-adjusted)**
Ernie Bot	83 (50.3)	8 (34.8)	8 (34.8)	14 (60.9)	13 (56.5)	26 (60.5)	7 (31.8)	7 (87.5)	0.080
ChatGLM	77 (46.7)	12 (52.2)	9 (39.1)	9 (39.1)	7 (30.4)	26 (60.5)	7 (31.8)	7 (87.5)	0.104
SparkDesk	58 (35.2)	8 (34.8)	8 (34.8)	6 (26.1)	9 (39.1)	14 (32.6)	7 (31.8)	6 (75)	1.000
GPT-4	62 (37.6)	10 (43.5)	10 (43.5)	5 (21.7)	4 (17.4)	20 (46.5)	5 (22.7)	8 (100)	< 0.004

*P* values are Bonferroni-adjusted across the four primary omnibus χ^2^ tests (α_adj = 0.0125).

### 3.3 Qualitative analysis of LLM's response quality

The performance of LLMs was evaluated using three qualitative indicators: logical reasoning, ability to use internal information, and ability to use external information (Table 5).

**Table 5:** Performance of LLMs' responses in evaluation metrics.

**Evaluation metric**	**Ernie Bot**				**ChatGLM**			
	**Overall** ***n*** = **165**, ***n*** **(%)**	**Correct** ***n*** = **83**, ***n*** **(%)**	**Incorrect** ***n*** = **82**, ***n*** **(%)**	***P*** **-value**	**Overall** ***n*** = **165**, ***n*** **(%)**	**Correct** ***n*** = **77**, ***n*** **(%)**	**Incorrect** ***n*** = **88**, ***n*** **(%)**	***P*** **-value**
**Logical reasoning**								
Yes	162 (98.2)	83 (100)	79 (96.3)	0.120	165 (100)	77 (100)	88 (100)	—
No	3 (1.8)	0 (0)	3 (3.7)		0 (0)	0 (0)	0 (0)	
**Internal information**								
Yes	161 (97.6)	82 (98.8)	79 (96.3)	0.306	165 (100)	77 (100)	88 (100)	—
No	4 (2.4)	1 (1.2)	3 (3.7)		0 (0)	0 (0)	0 (0)	
**External information**								
Yes	129 (78.2)	63 (75.9)	66 (80.5)	0.300	113 (68.5)	50 (64.9)	63 (71.6)	0.226
No	36 (21.8)	20 (24.1)	16 (19.5)		52 (31.5)	27 (35.1)	25 (28.4)	
**Evaluation metric**	**SparkDesk**				**GPT-4**			
	**Overall** ***n*** = **165**, ***n*** **(%)**	**Correct** ***n*** = **58**, ***n*** **(%)**	**Incorrect** ***n*** = **107**, ***n*** **(%)**	***P*** **-value**	**Overall** ***n*** = **165**, ***n*** **(%)**	**Correct** ***n*** = **62**, ***n*** **(%)**	**Incorrect** ***n*** = **103**, ***n*** **(%)**	***P*** **-value**
**Logical reasoning**								
Yes	72 (43.6)	12 (20.7)	60 (56.1)	< 0.001	165 (100)	62 (100)	103 (100)	—
No	93 (56.4)	46 (79.3)	47 (43.9)		0 (0)	0 (0)	0 (0)	
**Internal information**								
Yes	72 (43.6)	12 (20.7)	60 (56.1)	< 0.001	165 (100)	62 (100)	103 (100)	—
No	93 (56.4)	46 (79.3)	47 (43.9)		0 (0)	0 (0)	0 (0)	
**External information**								
Yes	40 (24.2)	5 (8.6)	35 (32.7)	< 0.001	119 (72.1)	42 (67.7)	77 (74.8)	0.213
No	125 (75.8)	53 (91.4)	72 (67.3)		46 (27.9)	20 (32.3)	26 (25.2)	

Logical reasoning: ChatGLM and GPT-4 could provide logical explanations for every answer selection. However, some of the responses from Ernie Bot and SparkDesk lacked logical reasoning. In the case of Ernie Bot, the presence or absence of logical reasoning did not have a significant effect on the accuracy rate of response (*P* = 0.12). However, a significant effect was observed on the presence of logical reasoning in SparkDesk (*P* < 0.001), suggesting that logical reasoning may impact correctness rates.

Ability to use internal information: Both ChatGLM and GPT-4 utilized internal information in all question explanations. In contrast, SparkDesk provided significantly less internal information in its parses compared to the other three models. The presence or absence of internal information in the response significantly impacted the accuracy rate for SparkDesk (*P* < 0.001).

Ability to use external information: For Ernie Bot, 75.9% (63/83) of correct responses and 80.5% (66/82) of incorrect responses presented external information (difference of 4.6%; *P* = 0.30). Similarly, for ChatGLM, 64.9% (50/77) of correct responses and 71.6% (63/88) of incorrect responses contained external information (difference of 6.7%; *P* = 0.23). However, in SparkDesk, only 8.6% (5/58) of correct responses and 32.7% (35/107) of incorrect responses presented external information (difference of 24.1%; *P* < 0.001). There was a significant effect on the accuracy rate for SparkDesk depending on the presence of external information. For GPT-4, the difference was 7.1% (*P* = 0.21).

Among the reasons for incorrect responses in the four LLMs, informational errors were the most common, followed by instances where both logical and informational errors occurred at the same time (Table 6).

**Table 6:** Statistical analysis of the reasons behind incorrect responses.

**Error type**	**Ernie Bot**		**ChatGLM**		**SparkDesk**		**GPT-4**	
	***n*** = **82**	***n*** **(%)**	***n*** = **88**	***n*** **(%)**	***n*** = **58**	***n*** **(%)**	***n*** = **103**	***n*** **(%)**
Logical error	10	12.2	3	3.4	2	1.9	1	1
Information error	51	62.2	77	87.5	86	80.4	101	98
Logical and information errors	21	25.6	8	9.1	19	17.7	1	1

## 4 Discussion

### 4.1 Principal findings

Our results indicated that Ernie Bot performed the best in terms of scores and correctness, followed by ChatGLM. Both of them exceeded the passing score, demonstrating their expertise in TCM surpassed the passing threshold for CPE-TCM. SparkDesk and GPT-4 did not reach the passing threshold. This suggests that Ernie Bot and ChatGLM have the potential to be used in TCM and could serve various roles in the future.

Differences in AI accuracy across input languages may partially stem from the linguistic composition of its dataset, as LLMs often exhibit a preference for languages more aligned with their training data (27). ChatGPT performs best with English input, emphasizing the influence of language on its accuracy (28). Yu et al. (29) discovered that ChatGPT demonstrated medium accuracy in answering open-ended medical questions in Chinese, with an accuracy rate of 31.5%. Considering the disparities in English and Chinese inputs, it's clear that ChatGPT needs further improvements to handle medical questions in Chinese. Furthermore, the performance of GPT-4 did not meet expectations in this study, likely because it is a general-domain model with relatively limited training data in TCM. To enhance the interoperability of Chinese medical terminology within international contexts, previous research has attempted to map Chinese medical entities to the Unified Medical Language System (UMLS), which helps cross-lingual models better understand and align TCM-related concepts (30).

After applying the Bonferroni correction across the four omnibus χ^2^ tests, only GPT-4 retained a statistically significant difference in performance across disciplines (*P*_adj < 0.004), while the apparent subject-level variations in Ernie Bot and ChatGLM (*P*_adj = 0.080 and 0.104, respectively) were no longer significant. This finding indicates that the previously observed heterogeneity for these two models may have been partly attributable to multiple testing and should therefore be interpreted with caution. The remaining subject-specific difference in GPT-4′s performance suggests that even the most advanced general-purpose LLM still faces challenges in mastering certain specialized areas of TCM knowledge, highlighting the importance of domain-specific fine-tuning.

These four LLMs did well in responding to the questions on the medical humanistic spirit, possibly because answering such questions does not necessitate specialized medical knowledge or clinical experience. In TCM education, students first learn foundational subjects before moving on to clinical subjects. The ability to effectively address clinical questions relies, in part, on mastering these fundamentals and applying them correctly. Ernie Bot showed a higher accuracy rate in answering questions about Pharmacology (foundational subjects) and Internal Medicine of TCM (clinical subjects), while ChatGLM and GPT-4 performed well in Internal Medicine of TCM. Consequently, in the present study, the remarkable performance of LLMs in Internal Medicine of TCM (clinical subjects) gave a huge impression. This may be because the Internal Medicine of TCM questions were mostly presented in case formats, where LLMs showed greater proficiency in extracting and processing internal information. Subject-level accuracy illustrates the suitability of various models for distinct domains, providing invaluable guidance for users seeking to choose a model tailored to their specific requirements (31).

In the qualitative analysis of responses, (1) In terms of logical reasoning ability, when the prompt specifically asked the LLMs to generate an answer parsing, Ernie Bot, ChatGLM, and GPT-4 produced more context-focused answers, which were better representations of the deductive reasoning process. SparkDesk had 56.4% of answers lacking logical reasoning. Meanwhile, the accuracy rate of responses showing logical reasoning exceeded those without it. This suggests an urgent need for SparkDesk to improve its deductive reasoning abilities to increase accuracy rates. (2) In terms of the ability to use internal and external information, Ernie Bot, ChatGLM, and GPT-4 incorporated internal information in over 95% of their answer parses, with over 60% integrating external information. The frequency of correct answers with external information did not significantly surpass that of incorrect answers. This suggested that although LLMs could connect questions to extra data, such information did not substantially aid in making the correct choice.

The causes of incorrect responses were categorized into three groups: logical errors, information errors, and both types of errors at the same time. General-domain LLMs were mainly trained on widely accessible public datasets, which might have limitations due to the lack of training data specific to professional knowledge within specialized domains (32). When confronted with issues necessitating domain-specific knowledge, general-domain LLMs can be significantly illusory, often resulting in factual fabrications (33). It closely aligns with the findings of the present study, where informational errors were identified as the most prevalent factor. Hence, prioritizing data selection and filtering from pre-trained corpora to gather high-quality TCM knowledge data is crucial. Moreover, considering the intricate terminology in TCM, improving the proficiency of LLMs in conducting expert analyses on TCM matters could be achieved through the selection of instruction-tuning data.

In this study, the evaluation of four LLMs' performance in the CPE-TCM revealed that Ernie Bot and ChatGLM were able to pass the exam. Furthermore, over 95% of responses demonstrated logical reasoning and internal information. The majority of responses also presented external information, indicating that they could justify the response in most cases by demonstrating logical reasoning and contextual information. Ernie Bot and ChatGLM could process and comprehend natural language inputs by leveraging their broad pre-trained knowledge to deliver coherent and well-parsed responses. As a result, the superior performance and accuracy of ChatGLM and Ernie Bot imply a greater proficiency in addressing TCM exam questions. This offers preliminary data that they can lead to integrated TCM applications, showcasing their enormous potential as TCM support tools. The knowledge accuracy and interpretive ability of SparkDesk and GPT-4 need further improvement.

### 4.2 Potential application of LLMs in TCM education

The logical reasoning and contextual comprehension demonstrated by LLMs highlight their potential as valuable tools in TCM education (18). LLMs can serve as “virtual assistants” to support various educational needs—such as generating multiple-choice questions, offering personalized feedback, simplifying complex concepts, and assisting in diagnostic training and prescription writing (34). Their ability to process nuanced language input and integrate background knowledge allows them to contribute meaningfully to knowledge consolidation and clinical thinking.

However, the deployment of LLMs in education requires caution. All outputs must be validated to ensure the safe and accurate dissemination of TCM knowledge, particularly given its specialized terminology and rule-based logic. While previous research has broadly envisioned LLMs as supportive tools, further specificity is needed to guide practical implementation in real-world teaching.

To address this gap, we propose stage-specific application scenarios that align LLMs functionalities with the core competencies of each phase in TCM education. These scenarios are designed to be actionable, verifiable, and conducive to educational outcomes.

### 4.3 Application scenarios of LLMs aligned with the stages of TCM education

Building upon prior research on the use of LLMs in medical education, we further integrate the staged characteristics of TCM training and its unique pedagogical components—such as syndrome differentiation, formula composition, and rule-based prescription logic—to propose structured and practical application pathways.

1. Chinese postgraduate examination stage

During preparation for CPE-TCM, LLMs can be deeply embedded into intelligent assessment and adaptive learning systems to facilitate high-level competence evaluation and personalized learning guidance.

- Generation and analysis of X-type questions

X-type questions (multiple-choice questions with multiple correct answers) are the most discriminative item type in CPE-TCM. LLMs can automatically generate such questions that examine the logical interconnections among multiple knowledge domains—including basic theories, Chinese materia medica, formulas, and internal medicine—and provide detailed reasoning for each option. This allows students to engage in horizontal comparison across subfields, deepening comprehension and memory.

- Error tracing and knowledge mapping

Unlike traditional “question–answer–explanation” feedback, LLMs can map incorrect responses to specific knowledge deficiencies (e.g., incompatibility rules, misinterpretation of pathogenesis) and trigger personalized review plans. This enables an adaptive learning loop of “error diagnosis—spaced repetition—reinforced training.”

A typical implementation scenario may include the following stages:

- Day 0: Initial attempt and immediate feedback

Students complete an LLM-generated X-type question. The system provides reasoning for each option and maps any incorrect choices to relevant weak points (e.g., “insufficient ability to differentiate between Qi deficiency and Yang deficiency” or “unclear understanding of the therapeutic functions of formulas”).

- Day 1: Rule reinforcement

The system pushes targeted microlearning content (e.g., comparison charts, mnemonic summaries) and focused discriminative exercises to strengthen key principles and correct fundamental misconceptions.

- Day 3: Knowledge integration and transfer

LLMs generate new questions linking the previously weak concepts with related domains (e.g., combining herbal properties, formula compatibility, and internal medicine pathogenesis), testing the learner's ability to apply and transfer knowledge to new contexts.

- Day 7: Contextualized comprehensive testing

The system presents a simulated clinical case requiring the learner to complete the entire reasoning chain—from syndrome differentiation to formula composition—thereby verifying whether fragmented knowledge has been internalized and integrated.

2. Standardized residency training stage

At the stage of standardized residency training, LLMs can serve as virtual case simulators and prescription audit assistants, enhancing clinical reasoning ability and medication safety awareness.

- Case variant generation integrated with SP training

Prior studies have shown that LLM-based virtual patients can effectively improve medical trainees' diagnostic and communication skills (35). Based on standardized patient (SP) scripts, LLMs can automatically generate diverse case variants by modifying syndrome patterns, tongue and pulse manifestations, or comorbid conditions. This enables multi-scenario training for syndrome differentiation. For example, a patient with a “spleen deficiency” pattern may be supplemented with a “current use of ibuprofen” context to test whether the trainee can recognize the adverse gastrointestinal risks of NSAIDs, thereby reinforcing awareness of medication safety.

Virtual patient interaction

LLMs can simulate natural patient communication, including realistic language style and emotional responses (35). The model dynamically adjusts dialogue pace and information disclosure depth based on the completeness and logic of the trainee's questions, improving the ability to collect the “Four Diagnostic Methods” (inspection, listening/smelling, inquiry, and palpation). Meanwhile, it can analyze the appropriateness of the trainee's diagnosis and prescription, identify logical flaws or safety warnings (e.g., *formula–pattern mismatch, pregnancy contraindication*), and provide instant, context-aware feedback.

- Prescription audit assistance

After the virtual consultation, trainees submit herbal prescriptions, which are automatically reviewed by the LLM to identify potential risks and provide revision suggestions to improve prescription safety and compliance.

Overall, LLMs can support the development of an integrated TCM Clinical Reasoning Training Platform that combines virtual and physical learning components:

Syndrome evolution simulation

Starting from a prototype syndrome pattern (e.g., *liver qi stagnation with spleen deficiency*), the model can generate evolved patterns such as *liver fire transformation* or *qi–yin deficiency*, automatically adjusting inquiry content and physical findings to train students' diagnostic and differentiation skills.

Symptom interference design

LLMs can deliberately obscure or disguise primary symptoms to test diagnostic accuracy—for instance, in a case of *cardiac blood stasis pattern*, an SP may primarily report “epigastric discomfort,” while the key symptom of “chest oppression” is downplayed, evaluating the trainee's ability to identify the core pathogenesis through detailed inquiry.

Contextualized prescription feedback

Upon prescription submission, the LLMs cross-references patient history and syndrome pattern, flags potential risks, and cites classical sources or modern clinical guidelines to provide evidence-based modification suggestions.

By aligning these LLM-driven functions with the progressive stages of “cognitive learning—clinical reasoning—safe practice” in TCM education, a systematic and intelligent support framework can be established. Future pilot programs could be conducted in small teaching cohorts to verify feasibility, accuracy, and educational outcomes, thereby providing an evidence-based pathway toward the intelligent transformation of TCM education.

### 4.4 Limitations

This study had the following limitations. First, CPE-TCM evaluated the basic competence of TCM students comprehensively but did not include a more detailed specialist assessment. Furthermore, LLMs are rapidly evolving, with their architectures and parameters continuously optimized over time, which poses objective constraints on reproducing identical experimental conditions. Given the inherent stochasticity of model generation, this study reports single-run results as representative performance estimates within each model version. Future research could conduct multiple independent trials on fixed model versions to obtain more robust statistical conclusions.

## Data Availability

The datasets presented in this study can be found in online repositories. The names of the repository/repositories and accession number(s) can be found below: https://doi.org/10.6084/m9.figshare.28682180.v3 the dataset is publicly available on Figshare.
